# The association between Solar Lentigines and Type-2 Diabetes

**DOI:** 10.22088/cjim.8.4.317

**Published:** 2017

**Authors:** Bobak Moazzami, Niloofar Razavi, Mansour Babaei, Motahareh Haghparast, Mohammad Ali Bayani

**Affiliations:** 1 Student Research Committee, Babol University of Medical Sciences, Babol, Iran.; 2 Department of Rheumatology, Ayatollah Rouhani Hospital, Babol University of Medical Sciences, Babol, Iran.; 3 Department of Internal Medicine, Babol University of Medical Sciences, Babol, Iran.

**Keywords:** Diabetes mellitus, Lentigo, Skin manifestations, Skin lesions

## Abstract

**Background:**

Limited information exists between the associations of diabetes mellitus (DM) and solar lentigo (SL); a benign, common skin lesion characterized by hyperpigmented macules.

**Methods:**

This study was conducted on 90 patients diagnosed with DM and their age and sex matched controls who were referred to the Departments of Endocrinology at Babol University of Medical Science in Northern of Iran from January 2013 to December 2015. All demographic data including age, gender, occupation, family history of DM, estimated average sun exposure and presence of skin lesions were collected and analyzed.

**Results:**

Presence of SL was significantly higher among patients with DM compared to controls (61.9% vs 33.6%, P<0.001). After adjusting for age, sex and sun exposure rate, results indicated that DM was independently associated with the presence of SL (p=0.002). Stratifying patients based on gender, indicated that DM was significantly associated with SL in females (p=0.03), but not in male patients (p=0.06).

**Conclusion:**

The findings of the present study indicate that DM is an independent risk factor for the occurrence of SL lesions. These findings could represent a novel association between DM as a chronic oxidation state and SL as an early sign of aging.

**D**iabetes mellitus (DM) is a multisystem disease and a major endocrine cause of morbidity and mortality around the world (1). DM can cause a variety of complications including microvascular (such as retinopathy, nephropathy and neuropathy) and macrovascular complications, especially in the skin (2-4). Previous studies have reported the frequency of diabetes-associated skin manifestations ranging between 30% to 96% during the course of disease (4-7). The knowledge regarding these skin disorders may be used as a first indicator for patient's metabolic status or it may be the only presenting symptom in undiagnosed diabetes (8). Although the pathogenesis of most diabetes-associated skin conditions remains unknown, abnormal carbohydrate metabolism, atherosclerosis, microangiopathy, neuron degeneration, and impaired host mechanism have been proposed as potential mechanisms (9). Solar lentigo (SL) is a benign common skin lesion which is characterized by hyperpigmented macules and mostly induced by chronic sun exposure (10). These lesions are mostly seen in middle aged or elderly people and are frequently present in sun-exposed areas such as the face and dorsal of the hands (11). While the mechanisms underlying the development of these lesions remain unclear, accumulation of advanced glycosylation end-products have been shown to contribute to the development of these lesions (12-13). Whether these SL lesions are associated with DM has not been investigated. Therefore, in the present study, we aimed to investigate the relation between type-2 diabetes as a chronic oxidative state and SL as an early sign of aging.

## Methods

The present study was conducted on patients who referred to the Department of Endocrinology Babol University of Medical Sciences northern Iran from January 2013 to December 2015. Ninety patients with diabetes aged 30-50 (14) were included as the cases. The control group consisted of 92 healthy and non-diabetic subjects who were matched by sex and age. The exclusion criteria were as follows; patients younger than 18 years of age, who had DM for 1 year or less and gestational DM.

An informed consent was obtained from all patients. All data were collected prospectively and systematically including age, gender, family history of DM, occupation and estimated average sun exposure. Patients were asked to estimate their average sun exposure in different seasons of a year (table 1). Then, the patients were categorized into 3 groups based on the amount of daily sun exposure including low exposure (<2 hours), medium exposure (2<hours<4) and high exposure (>5 hours) (table 1). The mean average of daily sun exposure was subsequently calculated. Participants were then classified as low exposure if exposure time was between 1 to 2 hour per day, intermediate around 2 to 4 hours daily and high for more than 5 hours day to day (table 1). 

All patients in both groups underwent a complete physical examination including detailed dermatological evaluation by a dermatologist. Essential microbiological and histopathological investigations of cutaneous lesions were carried out to confirm the diagnosis of SL. The size of the skin lesions on the dorsum of the hands were measured and categorized into 3 groups including small (S, <2mm), medium (M, 2mm-5mm) and large (L, > 5mm). The total number of lesions were also classified into two groups: few (number of macules less than 3) and high (number of macules 4 or more). This study was approved by the Ethics Committee of Babol University of Medical Sciences. All statistical analysis was performed using the SPSS software Version 17. A p-value of less than 0.05 was considered significant.

## Results

A total of 182 patients (78 males, 104 females) from 2 medical centers were enrolled in the study between January 2013 and December 2015. Among these patients, 90 were diagnosed with DM as the case group and 92 subjects in the healthy control group matched by age and sex were evaluated. The most common age group in this study was between 45 to 50 years (52.7%). A positive family history of diabetes mellitus was more common in cases versus controls (table 1). 

**Table 1:** Baseline characteristics of DM and matched control subjects

**Characteristic**	**Controls** **(N=92)**	**Cases** **(N= 90)**	* **P-value**
Age (mean ± SD)	43.53±4.63	44.54±4.28	NS
**Sex**			
Male Female	42 (45%) 49 (53.2 %)	36 (40%) 55 (61.1%)	NS
Family history of diabetes	21 (23.1%)	70 (76.9%)	P<0.001
Sun exposure rate			
Low exposure	82 (78.8%)	38 (48.7%)	NS
Medium exposure	2 (1.2%)	8 (10.3%)	NS
High exposure	20 (19.2%)	32 (41% )	NS
Presence of solar lentigo	33 (33.6%)	52 (61.9%)	P<0.001

NS: not significant

* P < 0.05 is considered to be significant.

**Figure 1:**
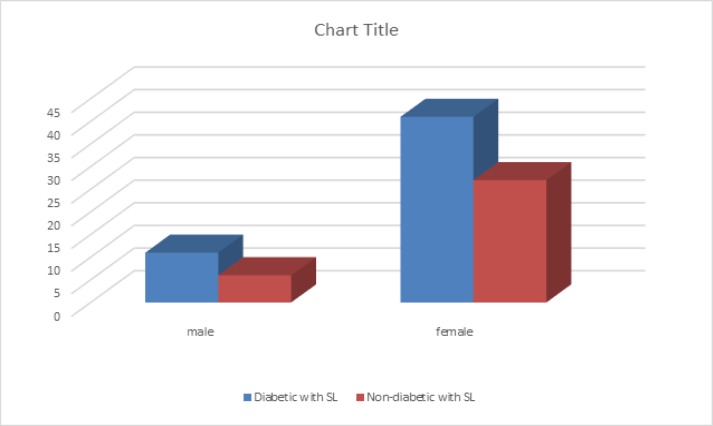
The frequency of patients diagnosed with diabetes mellitus in association with solar lentigo

Presence of solar lentigo was significantly higher among the patients diagnosed with DM, compared to controls (p<0.001) (figure 1). 

Among the diabetic subjects, the female gender was associated with the higher rate of SL (76% vs 31%, p<0.001). Data suggested that the frequency of exposure rate to sun was significantly higher in males than females (p<0.001). Regardless of the presence of diabetes, the existence of SL was significantly higher in diabetic female subjects compared to diabetic male subjects (p<0.0001). While the presence of SL was more prevalent in diabetic male subjects (64.7%) compared to non-diabetic males (35.2%), it was not statistically significant which could be partly due to the fewer number of subjects included in the study (p=0.21). After adjustment for age, sex and sun exposure rate, the presence of SL among subjects was analyzed. The results indicated that DM was independently associated with the presence of SL (p=0.002). Stratifying patients based on their gender indicated that DM was significantly associated with SL in female (p=0.03) but not in male patients (p=0.06).

## Discussion

The results of the present study indicate that the presence of SL was significantly higher among patients diagnosed with DM, compared to controls. After multivariable analysis, our results demonstrated that DM was independently associated with SL. While previous studies have shown that the incidence of cutaneous manifestation are higher among DM patients compared to general population (4-7), the association between DM and SL has not been previously investigated. The present study is the first report to describe the effects of impaired glucose metabolism on skin manifestations and the possible link between DM and the occurrence of SL.

SL also known as senile lentigo or liver spots, are hyperpigmentary disorders more commonly seen in older persons. It is considered to be a hallmark of photodamage and aging process. It is well-known that these spots occur mainly because of chronic ultraviolet (UV) exposure. Exposure to UV radiation results in increased melanin production and may lead to the development of these hyperpigmented lesions. This increase in melanin production is regulated by interactions between melanocytes and neighboring cells, such as keratinocytes or fibroblasts (15-16). In addition to fibroblasts, endothelial cells have shown to have a major role in pigmentation (20-21). There is strong evidence that suggests UV exposure can elicit angiogenesis and hyperpermeability of blood vessels in the dermis suggesting that microvasculature can play a role in the development of UV-associated pigmentary disorders such as SL (17-18). 

Besides the UV induced microvascular pathology, hyperglycemia has also shown to target vascular endothelial cells, playing a major role in the pathogenesis of diabetic macroangiopathy (19-21) Neuropathy, nephropathy, and retinopathy are all examples of microvascular complications of DM where the extension of glycemic control and duration of disease correlate with the extent of microangiopathy. The altered vascular endothelial function caused by abnormal carbohydrate metabolism can also be implicated in the etiology of cutaneous diabetic complications (21-23). This could partly explain the higher association between the SL lesions seen in our study among DM patients. 

The mechanisms of development of SL among patients with DM is unknown. Aging is shown to be independently associated with the development of SL. It has been shown that the cumulative amount of ultraviolet (UV) exposure, plays a role in the progression of SL (24, 25). The repeated UV exposure has shown to up regulate the inflammatory and fatty acid metabolism-related genes and therefore contribute in the induction of SL (25). Furthermore, recent studies have indicated that other environmental factors may also contribute to the development of SL. Our findings indicate that DM and impaired glucose metabolism are independently associated with SL development. Though the mechanisms for this association were not investigated in the present study, accumulation of advanced glycosylation end-products have shown to alter the structural properties of tissue proteins and reduce their susceptibility to catabolism (26). 

The major limitation of the present study is the relatively low number of subjects which prevented any subgroup analysis to determine the influence of variables such baseline demographics including age or the duration and stage of SL. Therefore, the findings of the present study should be interpreted in the context of its limitations. Further studies are required to elucidate the possible mechanisms of this association.

In conclusion, the findings in the present study have shown a novel relation between DM as a chronic oxidation state and SL as an early sign of aging. 
